# The Effect of Cannabidiol on the Viability and Anticancer Properties of Human NK Cells: Consequences for Therapies

**DOI:** 10.3390/biom16101491

**Published:** 2026-10-14

**Authors:** Cecilia Gutiérrez-Iñiguez, Miguel Olivas-Aguirre, Igor Pottosin, Oxana Dobrovinskaya

**Affiliations:** 1 Laboratory of Immunology and Ionic Transport Regulation, Biomedical Research Centre, University of Colima, Av. 25 de Julio #965, Villas de San Sebastián, Colima 28045, Mexico; cgtziniguez@gmail.com; 2 Laboratory of Cancer Pathophysiology, University Center for Biomedical Research, University of Colima, Colima 28045, Mexico; molivas@ucol.mx or miguel.olivas@secihti.mx; 3 Secretariat of Science, Humanities, Technology and Innovation (SECIHTI), Researchers for Mexico Program, Mexico City 03940, Mexico

**Keywords:** cannabidiol, cytotoxicity, mitochondrial permeability transition pore, NK cells

## Abstract

The palliative and anti-cancer properties of CBD are attracting increasing attention. We have examined the dose-dependent effects of CBD on the viability and antitumor properties of human NK cells. CBD killed NK cells with an apparent IC_50_ of 77 μM at 24–48 h of incubation. Concentrations up to 10 μM caused neither cell death nor a significant decrease in metabolic activity, whereas concentrations above 30 μM provoked cell death. The antagonist of CB_1_ partly reverted CBD cytotoxicity, whereas the antagonist of CB_2_ was inefficient. Cyclosporin A (CsA), an inhibitor of the mitochondrial permeability transition pore, mPTP, strongly suppressed the CBD cytotoxicity. CBD produced the CsA-sensitive depolarization of mitochondrial membrane potential in leukemic Jurkat cells, with a lesser effect on NK cells. The cytotoxicity of NK cells against Jurkat cells was evaluated in a 1:1 co-culture. Different donors displayed greatly variable NK cells’ degranulation, which was unaffected by a pre-treatment with up to 25 μM of CBD. NK cells from half of the tested donors displayed maximal cytotoxicity against target cells upon pretreatment with up to 25 μM of CBD, whereas in others, NK cytotoxicity decreased by 60%. Therefore, the appropriateness of including CBD in anticancer treatment should be carefully assessed based on the concentrations expected for palliative or antitumor effects.

## 1. Introduction

Cannabidiol (CBD), a non-intoxicating cannabinoid, as evidenced by preclinical data and clinical observations, interacts with the body’s endocannabinoid system and provides secondary relief for a variety of distressing symptoms like pain, anxiety, anorexia and neurological disorders (reviewed in [1,2,3]). Although the only FDA-approved pure plant-derived CBD is Epidiolex, which is indicated for severe, treatment-resistant childhood epilepsy [4], in many countries CBD is freely sold in a variety of formulations (liquid drops, capsules, pills, vapes, etc.), and frequently used by people, including cancer patients, for palliative purpose [3].

In recent decades, CBD has generated significant scientific interest due to its potential anti-tumor properties, which were evidenced by numerous preclinical in vitro and animal studies with several types of tumors (reviewed in [1,5,6,7]). It was demonstrated that CBD affects cancer cells through several pathways, arresting the cell cycle and preventing proliferation, reducing migration properties, and triggering cell death. Moreover, CBD was demonstrated to display synergistic effects with various chemotherapeutic drugs [8,9,10]. Based on this preclinical research, an active scientific discussion regarding the potential inclusion of CBD in anticancer protocols exists, both for supportive symptom management and as a direct antineoplastic compound. In this regard, important questions need to be addressed: how sensitive are different types of cancer to the toxic effects of CBD, and are cancer cells more sensitive to the toxic effects of CBD than healthy human cells, especially lymphocytes with anti-cancer activity, such as NK cells?

In our comparative study, cell lines derived from T-cell acute lymphoblastic leukemia (T-ALL) Jurkat, MOLT-3 and CCRF-CEM were significantly more sensitive to CBD toxicity than cells from other cancers: CBD at 30 μM eliminated about 90% of T-ALL cells, with only a moderate effect on the viability of B-cell acute lymphoblastic leukemia (Reh and RS4;11), breast cancer (MDA-MB-231 and MCF7) and adenocarcinoma (HeLa) cell lines, and without significant effects on the chronic myelogenous leukemia cell line K562 [11]. While the sensitivity of proliferating healthy T lymphocytes tested in vitro was comparable to that of leukemic T cells, resting T lymphocytes were more resistant and retained their ability to be activated after CBD treatment [11]. Freshly isolated human monocytes displayed a high sensitivity to CBD, so that at a 30 μM concentration the death toll was about 75% [12]. Although CBD was reported to induce immunosuppression in humans and animals (reviewed in [13]), studies with NK cells are very limited. Studies on the Wistar rat model demonstrated that CBD (16 μM)-induced lymphopenia involved B and T lymphocytes, but neither affected NK cells proliferation nor cytotoxic activity [14]. A recent study on isolated human peripheral blood mononuclear cells (PBMCs) reported a comparable toxic effect of CBD at a concentration of 20 μM on B, T and NK cells and monocytes. Cell death, however, was observed only in half of tested donors, and the effector activity of cells in respective PMBCs subpopulations has not been addressed [15].

NK cells are a type of innate immune cells capable of mediating the killing of tumor cells without the need for prior antigen sensitization. This makes NK cells a key component of the first line of defense against cancer cells. In recent decades, a novel immunotherapeutic strategy leveraging the anti-cancer potential of NK cells has been proposed [16,17,18,19]. Proposed sources of NK cells for immunotherapy include cells isolated from patients themselves or from healthy compatible donors, NK cells differentiated from hematopoietic stem cells (HSCs) or induced pluripotent stem cells (iPSCs), and established NK cell lines [19,20]. Importantly, peripheral blood-derived autologous or allogenic NK cells remain currently the traditional and most commonly used method for NK cell transfusion due to the following: (a) their proven safety based on decades of the clinical use of peripheral blood mononuclear cell-derived infusions; (b) the ease of access sourcing them from healthy adult donors via well-established workflow; (c) the high cytotoxicity of mature blood-derived NK cells possessing immediate potent killing capacity against tumor targets [18]. In this context, the question arises: to what extent do autologous or transfused NK cells retain their viability and functionality in patients using CBD to manage the side effects of chemotherapy? What potential cytotoxic and inhibitory effects might CBD exert on a patient’s NK cells if incorporated into a treatment protocol at higher doses as an anti-tumor agent?

CBD has multiple targets in leukocytes (including NK cells), such as canonic CB_2_ and, to a less extent, CB_1_ receptors, orphan G-protein coupled receptors, some TRPV vanilloid receptor channels, and PPAR-g receptors, among others (reviewed in [13,21]). However, the central mechanism of the CBD-induced death of human monocytes and T-ALL cells was shown to be independent of these receptors [11,12]. In both cases, the cell death was mediated by CBD targeting mitochondria and the opening of the mitochondrial permeability transition pore (mPTP), with a release of pro-apoptotic factors (cytochrome c) and cell death caused by mitochondrial dysfunction and increased ROS production [11,12]. In mouse models, CBD reduced the anti-cancer activity of NK cells acting via CB_2_ receptors [22]. To our knowledge, the mitochondrial targeting of CBD has never been addressed in studies of NK cells of either origin.

The objective of the present study was to estimate CBD concentrations toxic to NK cells from healthy human subjects, and to verify the involvement of CB_1_ and CB_2_ receptors and mPTP in cell death caused by CBD in NK cells. Another objective was to reveal how and which CBD concentrations that do not cause NK cell death affect their cytotoxicity against target tumor cells.

## 2. Materials and Methods

### 2.1. Reagents

CBD (Cat. #90081) was purchased from Cayman Chemicals (Ann Arbor, MI, USA). For the stock solution, 10 mg CBD/mL (32 mM) was dissolved in methanol (Cat. #154903; Merck KGaA, Darmstadt, Germany). Methanol was used as the vehicle control. The reference methanol concentration for vehicle control was 0.3% *v*/*v*, which corresponds to that applied with the highest tested CBD concentration (100 μM).

Cyclosporine A was from Merck (Cat. #30024). Synthetic antagonists of CB_1_, rimonabant hydrochloride (Cat. # SML0800), and CB_2_, AM630 (Cat. #AML0327) were from Sigma (St. Louis, MO, USA). These compounds were added 20 min prior to CBD exposure. Working concentrations were adjusted in accord with published dose–effect data.

All stock solutions were stored at −20 °C.

### 2.2. Cell Line and Culture Conditions

The human T-ALL cell line Jurkat (Clone E6-1, male, 14 years, ATCC^®^TIB™) was purchased from the American Type Culture Collection (ATCC; Manassas, VA, USA). Cells were grown in suspension in Advanced RPMI 1640 medium supplemented with 5% (*v*/*v*) heat-inactivated fetal bovine serum (FBS), 1% GlutaMAX™, 100 U/mL penicillin, 100 μg/mL streptomycin, and 10 mM HEPES (all from Gibco, Thermo Fisher Scientific, Grand Island, NY, USA). Cells were used for experimentation during the first 20 culture passages and maintained in a logarithmic growth phase by medium refreshment in a humidified incubator in an atmosphere with 5% CO_2_ at 37 °C.

### 2.3. Isolation and Purification of Primary NK Cells

Peripheral whole blood samples were collected from healthy adult donors after providing a concise explanation of the study’s purpose. All participants gave written informed consent in accordance with the ethical principles of the Declaration of Helsinki. The research protocol received approval from the Bioethics and Biosecurity Committee of the Biomedical Research Centre and the Faculty of Medicine at the University of Colima (Approval No. 2022-12) and adhered to federal regulations (Article 100, General Health Law). To maintain sterile conditions and ensure operator safety, all procedures were conducted inside a class II laminar flow biosafety cabinet. Whole blood was mixed in a 1:1 ratio with phosphate-buffered saline (PBS) at room temperature and layered over Ficoll Histopaque-1077 (Cat. #17144003; Cytiva, Uppsala, Sweden). in a 1:2 proportion. The samples were centrifuged at 1500× *g* for 40 min at 25 °C. Peripheral blood mononuclear cells (PBMCs) were collected, washed with PBS, and resuspended in complete RPMI-1640 medium. Natural killer (NK) cells were subsequently isolated using the NK Cell Isolation Kit (Cat. #130-092-657; Miltenyi Biotec, Bergisch Gladbach, Germany), following the manufacturer’s instructions. The purity of the isolated NK population (CD3^−^CD56^+^) was assessed by flow cytometry (FACS Canto II, BD Biosciences, Franklin Lakes, NJ, USA), using PE-conjugated anti-CD3 and FITC-conjugated anti-CD56 antibodies (300308 and 362546, respectively BioLegend, San Diego, CA, USA), achieving a final purity of 95.5 ± 1.77%. Purified NK cells were cultured in advanced RPMI 1640 medium supplemented with 5% heat-inactivated FBS, 100 U/mL penicillin, 100 μg/mL streptomycin, and 1% GlutaMAX™ in a humidified incubator at 37 °C with 5% CO_2_.

### 2.4. Trypan Blue Exclusion Test (Viability Assay)

Control and treated cells (1 × 10^6^ cells/mL) were seeded into 48-well culture plates and incubated for time periods ranging from 0 to 72 h. Following incubation, cells were gently resuspended to ensure homogeneity, and a 10 μL aliquot from each sample was mixed with an equal volume of trypan blue solution (Sigma-Aldrich, St. Louis, MO, USA Cat. #15250061) for viability assessment. A 10 μL portion of the resulting mixture was then loaded into a Neubauer hemocytometer to distinguish viable (unstained) from non-viable (blue-stained) cells. Cell viability counts were repeated in a minimum of four independent experiments. The resulting data were averaged and plotted as mean ± SEM.

### 2.5. Resazurin-Based Metabolic Assay

Cells (1 × 10^6^ cells/mL) were incubated for the determined periods (0–48 h) in a 96-well plate (180 μL/sample), separating them into two groups: with CBD (1–100 μM) and without CBD (vehicle). Subsequently, 20 μL of resazurin reagent (Tox-8, Sigma-Aldrich) was added to each well (for a total volume of 200 µL) and the mixture was further incubated for 4 h (37 °C). During incubation, non-fluorescent resazurin is metabolized by intracellular enzymes into resorufin, a highly fluorescent compound (Ex/Em max = 560/590 nm). Resorufin production by viable cells was estimated using a GloMax plate reader (Promega, Madison, WI, USA). Samples were excited at 525 nm, and the emitted fluorescence signal was recorded at 580–640 nm. The remaining medium fluorescence was subtracted. Data values were normalized to control and plotted as mean ± SEM for at least four independent experiments.

### 2.6. CD107a Degranulation Assay

The degranulation of NK cells in response to stimulation with target cells was assayed by flow cytometry (FACS Canto II) using CD107a Degranulation Assay. Freshly isolated NK cells were pretreated for 24 h with different concentrations of CBD or vehicle contained medium (control), washed, and mixed with target Jurkat cells in a 1:1 effector:target ratio in HBSS. Monensin (2 µM), a carboxylic ionophore that neutralizes the acidic pH of lysosomes, was added to the incubation solution to prevent the autophagic degradation of the endocytosed/trogocytosed anti-CD107a by constitutive protein recycling via target cells. After 4 h of incubation, anti-CD107a antibodies conjugated with FITC (Biolegend) were added, and samples were analyzed by flow cytometry. NK cells were first identified in the FSC-A/SSC-A dot plot based on their smaller size and lower granularity relative to target Jurkat cells, and subsequent analysis of the percentage of CD107a-positive cells was performed in this gated population.

### 2.7. Apoptosis/Necrosis Cell Death Assay in NK and Target Jurkat Cells

To assess CBD-induced cell death in NK cells, the Dead Cell Apoptosis Kit (Thermo Fisher Scientific, Cat. #V13241) was used, which allows for the discrimination of apoptotic and necrotic cells through dual staining with Alexa Fluor 488-conjugated recombinant annexin V and propidium iodide (PI), a nucleophilic marker, to establish necrosis. NK cells cultured in complete medium at a density of 1 × 10^6^ cells/mL were exposed to increasing concentrations of CBD (0–100 μM) for 24 h. Following treatment, cells were collected, counted, and centrifuged at 400× *g* for 5 min. The supernatant was discarded, and the resulting pellet was resuspended in annexin-binding buffer supplemented with 2 μL of annexin V-A488 and 1 μL of PI (200 μg/mL). Samples were incubated for 20 min in the dark at room temperature. After incubation, cells were analyzed by flow cytometry (FACS Canto II), using a 488 nm laser to excite both fluorophores—Alexa Fluor 488 (Ex/Em: 488/510 nm) and PI (Ex/Em: 535/617 nm).

To evaluate the cytotoxic effect of NK cells against target cells, CBD-pretreated NK and Jurkat cells were cocultured over 24 h in a 1:1 effector:target ratio. Then, cells were recollected, stained with Alexa Fluor 488-conjugated recombinant annexin V and propidium iodide (PI), as described earlier, and analyzed by cytometry. Jurkat target cells were first identified in the FSC-A/SSC-A dot plot based on their distinct size and granularity relative to NK cells, and all subsequent death analyses were performed on this gated population.

Data acquisition and analysis were performed using FlowJo software (version 7.0) to evaluate the contributions of early apoptotic (A488^+^/PI^−^), necrotic (A488^−^/PI^+^), and late apoptotic/necrotic (A488^+^/PI^+^) cells.

### 2.8. Determination of Mitochondrial Membrane Potential

CD56^+^ cells isolated from PBMCs were stained with the cationic fluorescent dye tetramethylrhodamine ethyl ester (TMRE; 200 nM, Ex/Em max = 555/582 nm; T669, Thermo Fisher Scientific) by incubating the cells for 30 min at room temperature (25 °C). After incubation, cells were centrifuged at 400× *g* for 10 min and washed with Hanks’ Balanced Salt Solution (HBSS; 143 mM NaCl, 6 mM KCl, 5 mM MgSO_4_, 1 mM CaCl_2_, 20 mM HEPES, 0.1% BSA, 5 mM glucose, pH 7.4, ~300 mOsm) to remove excess dye.

For spectrofluorometric analysis, stained cells (1 × 10^6^ cells/mL in HBSS) were transferred to a quartz cuvette and analyzed using a Hitachi F7000 spectrofluorometer (Hitachi High-Technologies Corporation, Tokyo, Japan). in time-series mode, with excitation at 555 nm and emission collected at 585 nm. During measurements, the cell suspension was maintained at 37 °C under continuous moderate stirring. Jurkat cells and CD56^−^ PBMC-derived cells were processed under identical conditions for comparison. Raw fluorescence data were exported to Excel and further processed and graphed using GraphPad Prism software.

To evaluate the effects of CBD on mitochondrial membrane potential after prolonged exposure, primary NK cells were incubated with increasing concentrations of CBD (5–60 μM) in 24-well plates for 24 h. After incubation, cells were harvested, centrifuged (400× *g*, 10 min), resuspended in HBSS, and transferred to custom-built imaging chambers with coverslip bottoms for live-cell analysis. TMRE retention was quantified by confocal microscopy (LSM 700, Carl Zeiss; black edition; Carl Zeiss Microscopy GmbH, Jena, Germany) using a 63× oil immersion objective. Images were acquired and analyzed with ImageJ software (version 1.54p; National Institutes of Health, Bethesda, MD, USA). Regions of interest (ROIs) were manually selected based on cell morphology, and TMRE fluorescence intensity was quantified. Data were subsequently analyzed and visualized in GraphPad Prism, and corresponding statistical analyses were performed.

### 2.9. Data Analysis and Statistics

In all experiments, at least three independent replicates from multiple donors were performed (*n* ≥ 3). Data were analyzed using GraphPad Prism 8.3 and are expressed as mean ± standard error of the mean (SEM). Statistical significance was assessed using one-way or two-way analysis of variance (ANOVA), followed by Tukey’s multiple comparisons tests, as appropriate.

## 3. Results

### 3.1. Assessment of CBD-Induced Cytotoxicity in NK Cells

**Figure 1:**
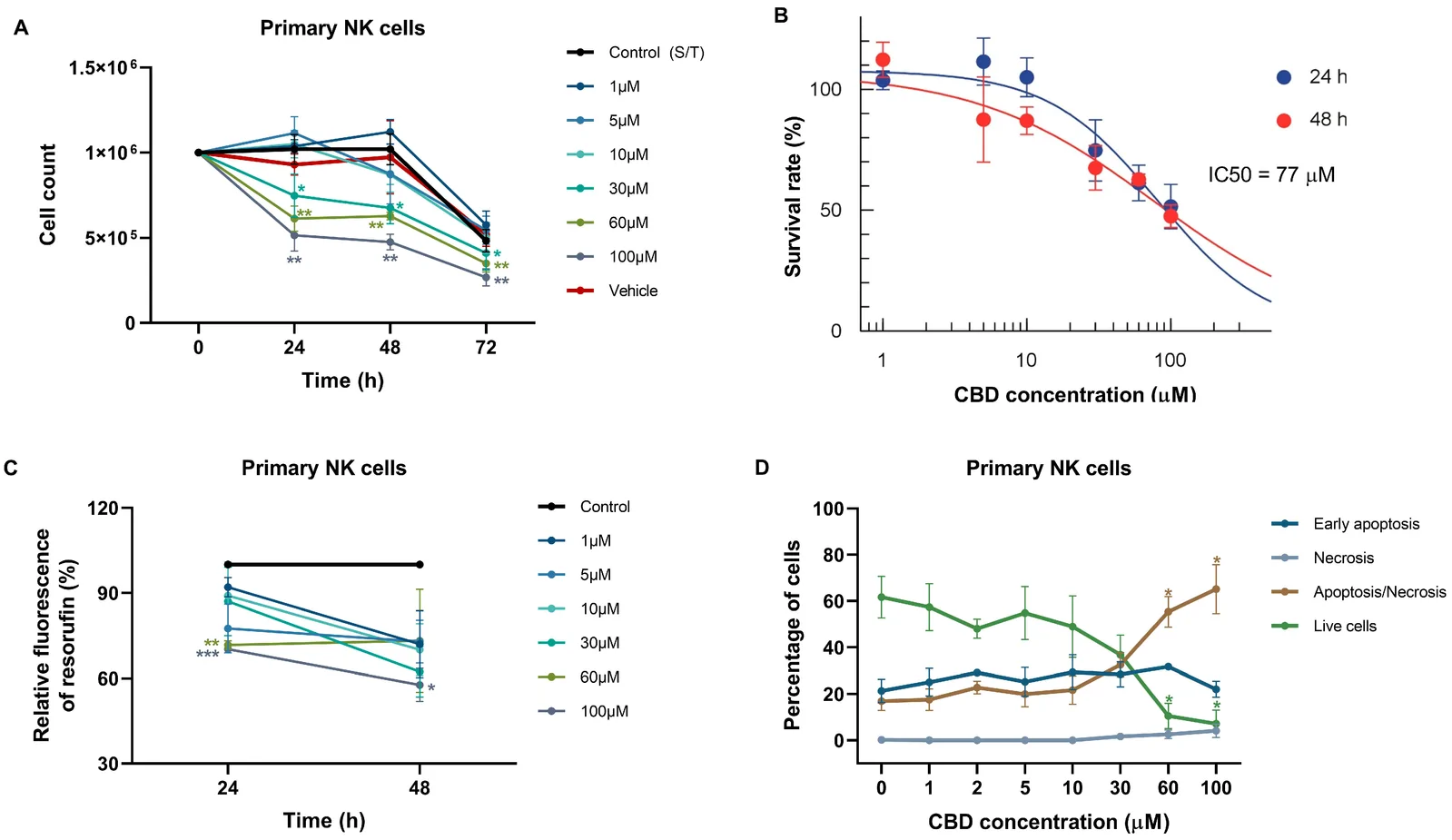
Cytotoxic effect of CBD on NK cell viability. (**A**) Live cell count (Trypan blue exclusion test) in NK cell cultures exposed to various concentrations of CBD (0–100 μM). Cell counts were assessed 24, 48, and 72 h after a single CBD treatment. Data reported as mean ± SEM (*n* = 4). Statistical significance (* *p* < 0.05; ** *p* < 0.01; Two-way ANOVA with Tukey post hoc test) relative to untreated control at the corresponding time point (24, 48, or 72 h) is indicated; time 0 was not used as the reference. (**B**) Half-maximal inhibitory concentration (IC_50_) determination. NK cell cultures were exposed to different CBD concentrations (0–100 μM), and IC_50_ values were determined at 24 and 48 h after CBD treatment by live cell count. (**C**) Cell viability, evaluated by resazurin-based metabolic assay, as a function of CBD concentration in NK cells at 24 h and 48 h of treatment. Data (resorufin fluorescence intensity) are normalized to vehicle-treated control and reported as mean ± SEM (*n* = 4) (* *p* < 0.05; ** *p* < 0.01; *** *p* < 0.001; Two-way ANOVA with Tukey post hoc test). (**D**) Cell death was evaluated by flow cytometry in NK cells treated with different concentrations of CBD for 24 h using annexin V-AF488/PI double staining. Annexin V − PI− represents live cells, Annexin V + PI− early apoptotic cells, and Annexin V − PI+ primary necrotic, whereas the double-stained population Annexin V + PI+ (DP) may include necrotic and late apoptotic ones. Data are mean ± SEM; *n* = 4. Statistical significance is relative to untreated control * *p* < 0.05; Two-way ANOVA with Tukey post hoc test.

Freshly isolated NK cells are notoriously short-lived in cultures in vitro. Although their long-term survival was reported to be improved in the presence of supportive cytokines like IL-2 or IL-15, prolonged in vitro incubation in the presence of IL-2/IL-15 was also reported as stimulating NK-cell proliferation and degranulation as well as NK-cell exhaustion [23,24]. Then, we avoided adding IL-15 and IL-2 to the culture medium, to prevent the premature activation and degranulation of NK cells prior to testing their sensitivity to CBD. Under these experimental conditions, cell survival and metabolic activity were maintained at a satisfactory level during the first 48 h after isolation, but not for 72 h (Figure 1A,C, Control and Vehicle), so all subsequent experiments were assayed at times not exceeding 48 h.

NK cell sensitivity to CBD was assessed using live cell count, a resazurin-based metabolic assay, and an apoptotic assay based on tracking changes in plasma membrane symmetry and integrity. As expected, CBD treatment in high concentrations (30–100 µM) resulted in a gradual concentration- and time-dependent reduction in the number of NK living cells (Figure 1A), with an apparent IC_50_ = 77 µM at 24 and 48 h of incubation (Figure 1B). The same IC_50_ values at 24 h and 48 h suggest that CBD exerts its peak inhibitory effect within the first 24 h of incubation, suggesting rapid cellular uptake and target engagement. We observed a significant decrease in metabolic activity in CBD-treated cell populations (Figure 1C), with a higher proportion of apoptotic and necrotic cells (Figure 1D). The increased proportion of dead cells (annexin V-single positive and double-positive) in the control untreated population, in comparison to the high cell viability evaluated by the Trypan blue exclusion test at this time point (Figure 1A), appears to be due to a high sensitivity of primary NK cells to mechanical disturbance during sample acquisition by cytometry, involving high hydrostatic pressure and hydrodynamic shear stress. Additionally, non-apoptotic phosphatidylserine externalization can be caused by NK cell activation triggered by mechanical stress.

### 3.2. Involvement of CBD Receptors in CBD-Induced Cell Death

**Figure 2:**
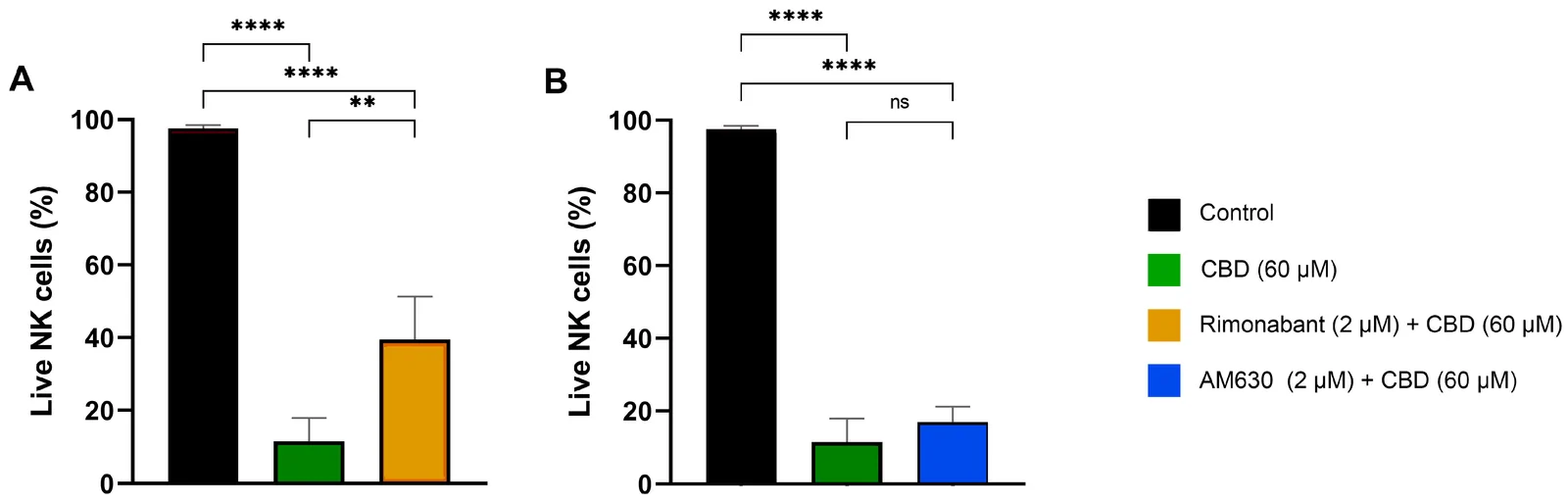
Impact of CBD receptor antagonists on NK cell viability in the presence of CBD. Data for the control group (black), cells treated with 60 μM CBD (green), and cells preincubated with pharmacological modulators of CB_1_ and CB_2_ receptors before CBD administration are presented. Preincubation concentrations were 2 μM of rimonabant (orange bar) and 2 μM of AM630 (blue bar). Data are presented as mean ± SD *n* = 4; ns, not significant; ** *p* < 0.01; **** *p* < 0.0001, One-way ANOVA with Tukey post hoc test). (**A**) CB1 antagonist rimonabant reverts CBD cytotoxicity in primary NK cells. Data for control group (black), cells treated with 60 μM CBD (green), and cells preincubated with 2 μM of rimonabant (orange bar) before CBD administra-tion are presented. Data are presented as mean ± SD *n* = 4; ** *p* < 0.01; **** *p* < 0.0001, One-way ANOVA with Tukey post hoc test). (**B**) CB2 antagonist can not reverts CBD cytotoxicity in primary NK cells. Data for control group (black), cells treated with 60 μM CBD (green), and cells preincubated with 2 μM of AM630 (blue bar) before CBD administra-tion are presented. Data are presented as mean ± SD *n* = 4; ns, not significant, **** *p* < 0.0001, One-way ANOVA with Tukey post hoc test).

Primary NK cells express CBD receptors CB_1_ and CB_2_, and their contribution to CBD-induced cell death has been described in several cellular models (reviewed in [1,21]). To investigate the involvement of these receptors in the mechanisms underlying the cytotoxic effect of CBD on primary human NK cells, cells were pre-incubated (for 20 min) with pharmacological blockers targeting these receptors, followed by the addition of CBD at a cytotoxic concentration (60 µM). After 24 h of incubation with CBD, we observed that the CB_1_ receptor antagonist rimonabant [25] was able to partially reverse the cytotoxic effect of CBD, in contrast to the CB_2_ receptor antagonist/inverse agonist AM630 [26] (Figure 2). Thus, the CB_1_ receptor, rather than the CB_2_ receptor, appeared to contribute to the cytotoxic mechanisms triggered by CBD in NK cells.

### 3.3. CBD Induced Mitochondrial Depolarization via mPTP Formation in NK Cells

**Figure 3:**
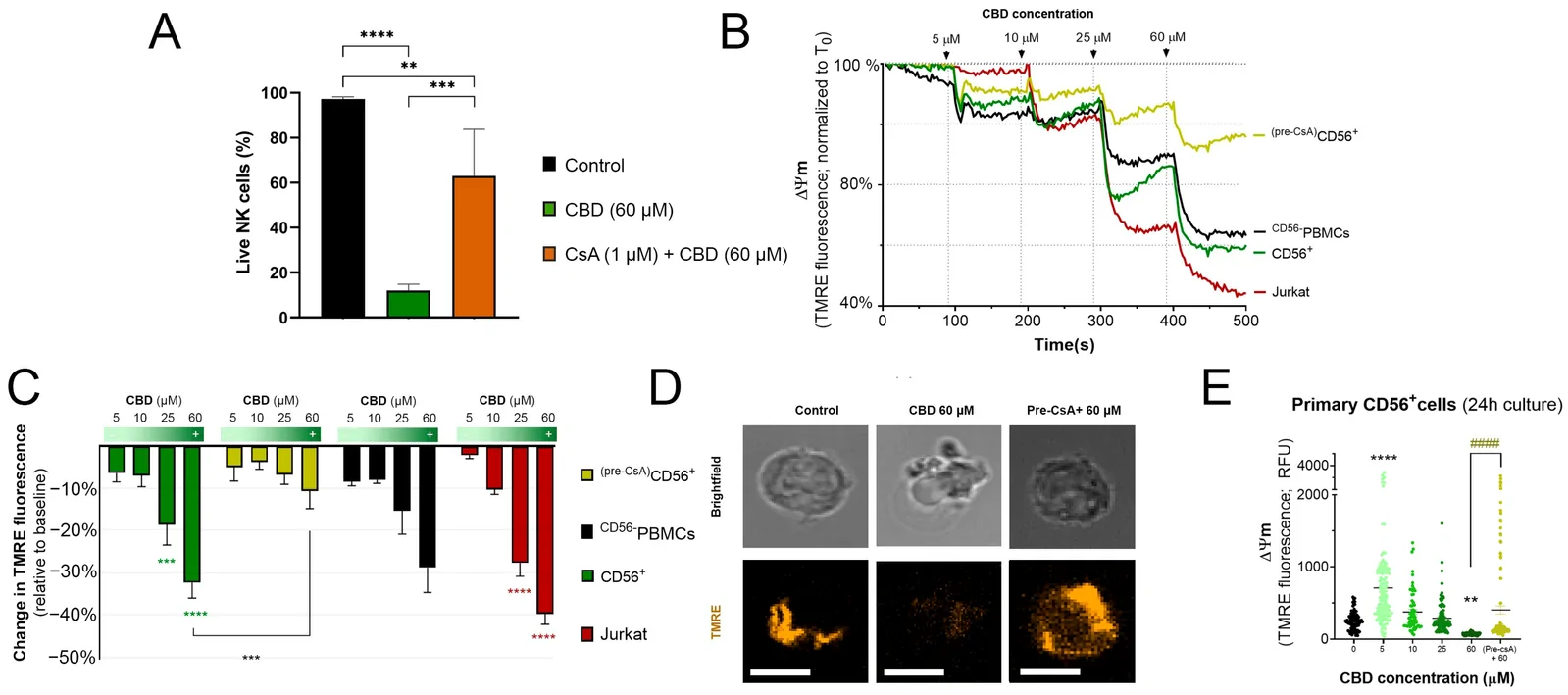
NK cells are resistant to CBD-induced mitochondrial depolarization. (**A**) CsA reverts CBD cytotoxicity in primary NK cells. Data for the control group (black), cells treated with 60 μM CBD (green), and cells preincubated with 1 μM CsA (orange) before CBD administration are presented. Data are presented as mean ± SD *n* = 4; ** *p* < 0.01; *** *p* < 0.001; **** *p* < 0.0001, One-way ANOVA with Tukey post hoc test. (**B**) Acute changes in mitochondrial membrane potential (TMRE relative fluorescence) in response to the sequential addition of CBD to the medium (arrows; final concentrations for each application are indicated). CD56^+^ cells (green), CD56^−^ PBMCs (black), CD56^+^ cells preincubated with cyclosporin A for 20 min (yellow), and leukemic Jurkat cells (red). Each trace represents the average of at least four independent experiments (*n* = 4). (**C**) Relative dissipation of mitochondrial membrane potential as a function of acute application of CBD at increased concentrations. A two-way ANOVA followed by Tukey’s multiple comparison test was used. Statistical significance is indicated as *p* < 0.001 (***) and *p* < 0.0001 (****). (**D**) Representative confocal micrographs of primary NK cells under control conditions, exposed to CBD (60 μM) or pretreated (20 min) with the mPTP inhibitor CsA (1 μM) prior to CBD exposure. Images were taken at 24 h in control conditions or after treatment. Scale bar: 5 μm. (**E**) Quantitative analysis of confocal micrographs showing mitochondrial membrane potential in primary NK cells after 24 h of exposure to CBD. Pretreatment with the mPTP inhibitor CsA (1 μM) reduced the loss of mitochondrial membrane potential induced by 60 μM CBD. Statistical analysis was performed using one-way ANOVA followed by Tukey’s multiple-comparisons test. Asterisks indicate significant differences versus the untreated control, whereas hash symbols indicate significant differences between treated groups. Statistical significance: ** *p* < 0.01; **** *p* < 0.0001; #### *p* < 0.0001.

As demonstrated by our group earlier, CBD provoked cell death in leukemic lymphocytes via a stable induction of the mitochondrial permeability transition pore mPTP. Cyclosporine A (CsA), a specific inhibitor of mPTP formation [27], significantly improved the viability in CBD-treated Jurkat cells [11]. Similar results were reported for human monocytes [12]. In the present study we have shown that CsA protected freshly isolated human NK cells against CBD cytotoxicity (Figure 3A).

Energized mitochondria display voltage difference (ΔΨm) across the inner membrane of −150 to −180 mV [28]. TMRE, a membrane-permeable fluorescent cation, is attracted by this voltage difference, such that TMRE fluorescence, which is proportional to the TMRE accumulation, is a measure of mitochondrial membrane potential level.

The sddition of 5 or 10 µM CBD did not induce a significant loss of ΔΨm in primary NK cells (Figure 3B, green trace). Exposure to 25 µM CBD caused a rapid drop in ΔΨm followed by its partial restoration. At 60 µM CBD the ΔΨm loss was significant and sustained over time. CBD-induced mitochondrial depolarization was substantially mitigated by the presence of CsA, a well-known inhibitor of mPTP formation (Figure 3B,C, yellow trace and bars). Leukemic lymphocytes showed a greater and irreversible ΔΨm loss at 25 µM CBD. At 60 µM CBD, the depolarization was further exacerbated and increased over time (Figure 3B,C, red trace and bars). CBD had a similar effect on non-NK (CD56^−^) PBMCs to that in NK cells (Figure 3B,C, black trace and bars). Thus, the CBD induction of mPTP is likely a common mechanism in different immune cells, while T-ALL cells display a lower ability to restore the mitochondrial potential.

To verify the adaptation of mitochondrial function to CBD treatment in time, we incubated NK cells with CBD (5–60 µM) for 24 h. TMRE staining after 24 h revealed a marked loss of mitochondrial potential only at 60 µM CBD (Figure 3D,E). Cells treated with 60 µM CBD displayed nuclear condensation, apoptotic body formation, and TMRE loss. These cell death features were significantly limited by CsA (Figure 3D, far right; Figure 3E). Of note, 5 µM CBD treatment over 24 h resulted in significantly increased TMRE retention (apparent hyperpolarization), which may be interpreted as an enhancement of the mitochondrial energization.

### 3.4. Impact of CBD on Degranulation of Primary NK Cells Following Stimulation by Target Cells

Upon the stimulation of cytotoxic NK cells by sensitive target cells, secretory granules/lysosomes rapidly fuse with the plasma membrane, causing the surface appearance of CD107a, which is a well-established indicator of NK-cell degranulation [29]. To determine whether CBD modulates this process, NK cells pretreated for 24 h with different concentrations of CBD were cocultured with Jurkat target cells (4 h, 1:1 ratio), and CD107a expression was quantified by flow cytometry.

**Figure 4:**
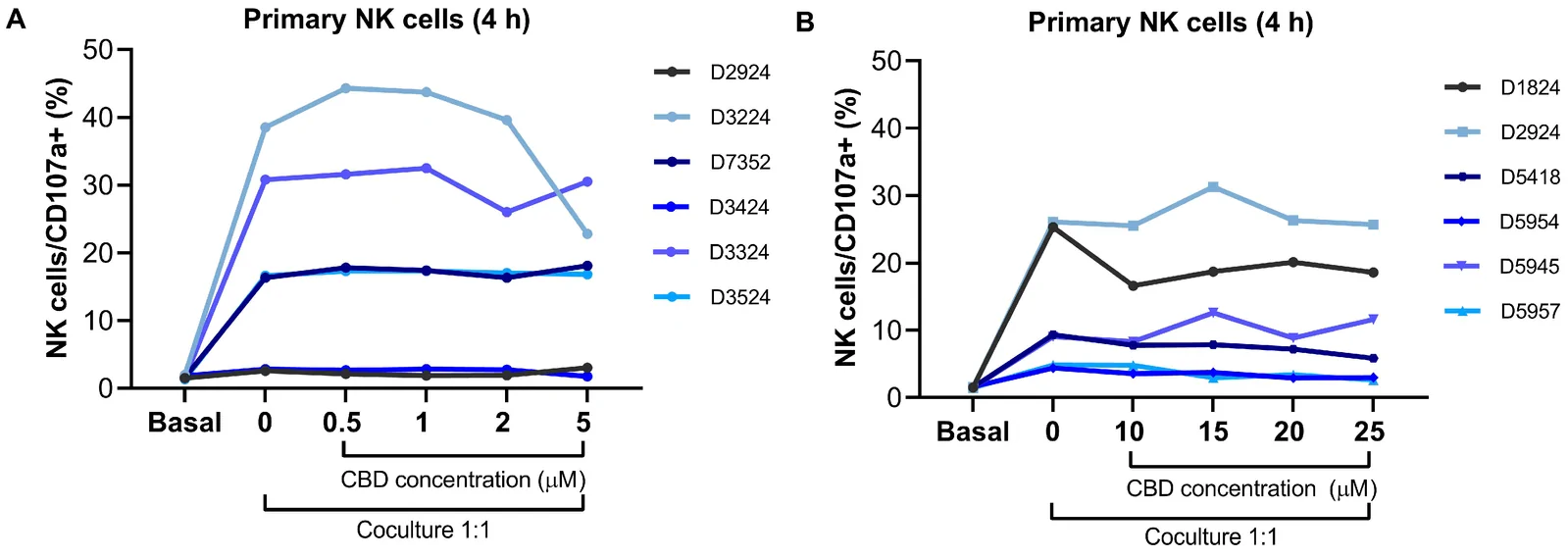
Degranulation of primary NK cells pretreated (24 h) with CBD in response to stimulation with target cells. (**A**,**B**) Individual donor data, showing the percentage of NK cells that externalized CD107a during 4-h co-culture with Jurkat cells (1:1 effector:target ratio).

First, NK cells did not demonstrate degranulation in monocultures without stimulation by target cells (Figure 4A,B, basal). Stimulation by Jurkat cells caused the degranulation of NK cells, but substantial heterogeneity in degranulation levels between donors was observed; some donors showed robust activation (30–40% of NK cells in the population responded in samples derived from D3224, D3324, D2924 (in B), and D1824), others exhibited moderate responses (≥10%; in D3524, D7352¸ D5418, and D5945), while several donors displayed weak or no activation (<10%; D5954, D5957, D2924 (in A), and D3424). Due to this heterogeneity, individual results for each donor are present (Figure 4A,B, 0 μM CBD). It was observed that preincubation with non-toxic concentrations of CBD (0.5–25 μM) did not alter NK-cell degranulation during a 4-h stimulation period in the majority of donors (10 out of 11 individual donors) (Figure 4A,B). Interestingly, the same donor (D2924) participated in two different experiments, and NK cells from his/her samples collected on different days demonstrated substantially different levels of degranulation in response to target cells. These data indicate that NK cell degranulation activity is a dynamic parameter and can vary from day to day.

### 3.5. Effect of CBD on the Cytotoxic Response of Primary NK Cells Against Jurkat Target Cells

**Figure 5:**
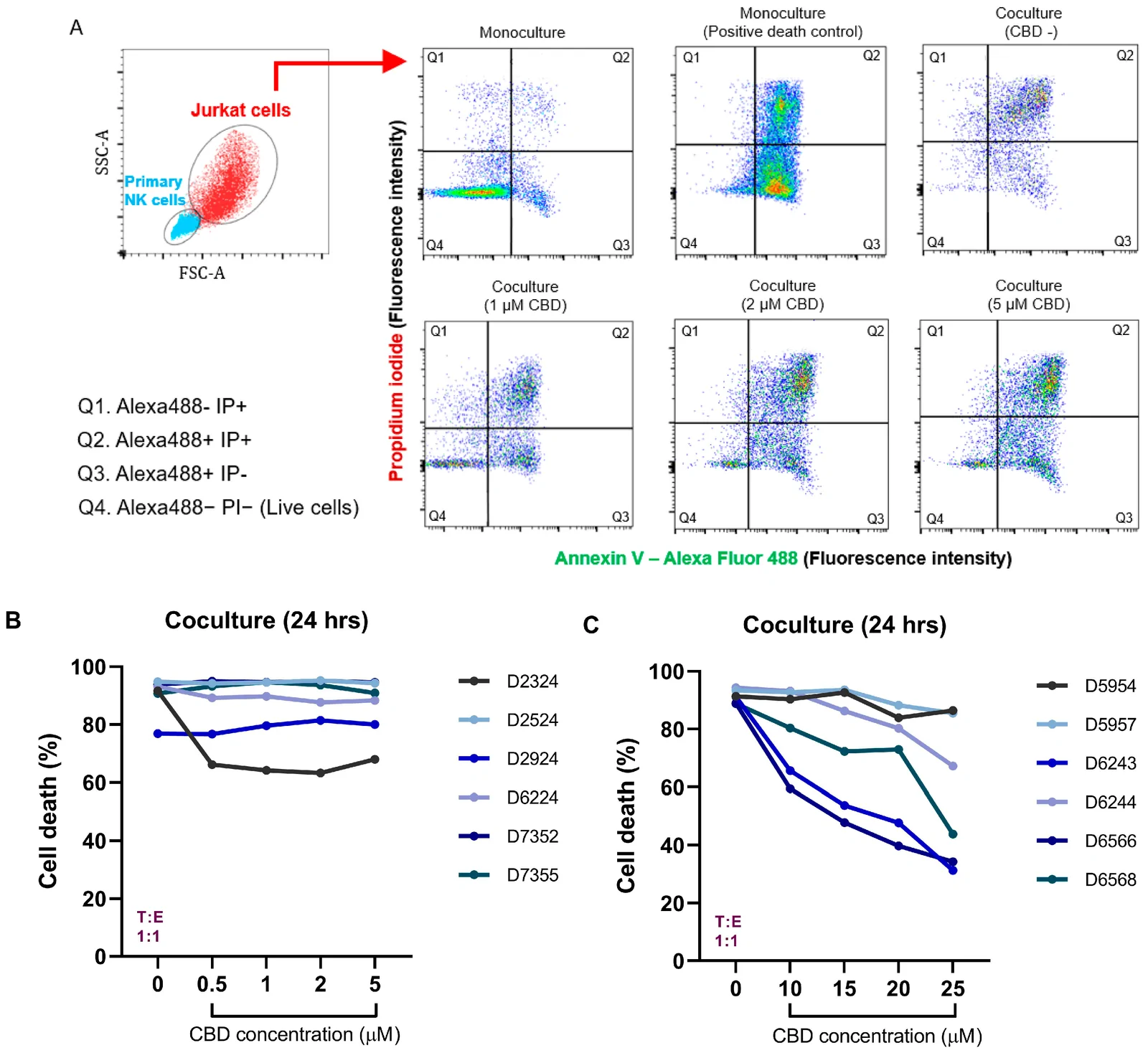
Cytotoxicity of NK cells pretreated with CBD against Jurkat cells. (**A**) Representative dot plots, showing Jurkat cell death after co-culturing for 24 h with NK cells previously pretreated with various concentrations of CBD. Cell death was evaluated by flow cytometry using Annexin V-AF488/PI double staining. In the FSC-A/SSC-A plot, cyan and red dots represent primary NK cells and Jurkat cells, respectively; the red arrow indicates the Jurkat cell population selected for analysis. In the Annexin V-AF488/PI plots, pseudocolors indicate increasing event density from blue through green and yellow to red. Green and red axis labels indicate Annexin V-AF488 and PI fluorescence, respectively. In every dot plot, the lower left quadrant represents Annexin V − PI− (live cells, Q4); in the lower right quadrant are Annexin V + PI− (early apoptotic) cells (Q3); Annexin V − PI+ (primary necrotic) cells are in the upper left quadrant (Q1), whereas the double-stained population Annexin V + PI+ (DP) in the upper right quadrant (Q2) represents dead cells, which may include necrotic and late apoptotic ones. (**B**) Percentage of target cell death in a 1:1 E/T co-culture. NK cells (donors D2324–D7355) were pretreated with CBD concentrations ranging from 0.5 to 5 μM. Jurkat cell death was evaluated after 24 h. (**C**) In a different set of experiments, NK cells from donors (D5954–D6568) were pretreated with CBD concentrations ranging from 10 to 25 μM.

The degranulation assay (surface mobilization of CD107a) measures the activation of NK cells and the release of cytotoxic granules, which does not necessarily provoke successful target cell death. It should be also taken into consideration that NK cells kill target cells through two mechanisms: granule exocytosis (the perforin–granzyme pathway) and granule-independent death receptor-mediated apoptosis (CD95/FasL extrinsic apoptotic pathway) [30]. Then, to prove the functional effector capability of NK cells to kill target cells, we determined the cell death of Jurkat cells co-cultured for 24 h with primary NK cells using an apoptosis/necrosis assay for flow cytometry (Figure 5). To assess the effect of CBD on NK cell cytotoxicity towards target leukemic cells, NK cells were pretreated for 24 h with various concentrations of CBD and then washed before coculturing with target cells to prevent the direct exposure of highly CBD-sensitive Jurkat cells to the drug. NK and Jurkat cells were then cocultured for 24 h, and the cell death of target cells was quantified by flow cytometry using Annexin V-A488 and propidium iodide. The treatment of a monoculture of Jurkat cells with etoposide (34 μM, 24 h) was used in each experiment as a positive control of cell death to ensure the staining procedure, resulting in 96–100% cell death. The analytical strategy for Jurkat cell death in co-cultures is described in Section 2 and illustrated in Figure 5A. Interestingly, the donors participating in these experiments did not demonstrate considerable inherent variability in baseline cytotoxicity (Figure 5B,C, 0 μM CBD). The pretreatment of NK cells with low CBD concentrations (0.5–5 µM) did not diminish their cytotoxicity toward target cells in most of the donors (four of five) (Figure 5B). In one out of five donors (D7355), the NK cytotoxicity was diminished by 40% in the presence of CBD. Higher concentrations of CBD (10–25 μM) had an inhibitory effect in a majority of donors, the extent of which varied considerably (Figure 5C). Two donors (D5954, D5957) showed no change in target cell-killing across the entire concentration range, where four donors exhibited a reduction in Jurkat cell death, with the magnitude of this decrease differing among individuals. In two donors (D6566, D6243), even 10 µM CBD reduced leukemic cell death by up to 60%, and the sublethal dose of 25 µM further decreased killing efficiency to approximately 30–40% of that in the control.

## 4. Discussion

NK cell-based immunotherapy using autologous and allogenic NK cells represents an emerging, innovative approach for enhancing cancer treatment efficacy and overcoming drug resistance [16,17,18,19]. Scientific discussions and active research are ongoing also regarding the role of CBD in anticancer therapy, either as a palliative agent or as a direct anti-cancer drug [1]. Careful evaluation is required to ensure that CBD does not interfere with the viability and anticancer properties of NK cells, to determine where the use of CBD is compatible with NK cell-based immunotherapy. Here, it is important to distinguish between relatively low CBD concentrations, used to manage pain, anxiety, stress and sleep disorder, and higher ones, used to suppress cancer cells.

The aim of the present work was to reveal the sensitivity of primary NK cells derived from apparently healthy donors to CBD. To our knowledge, there was a single study addressing the effect of CBD (20 μM) on the viability of human PBMCs subpopulations [15]. It was demonstrated that this single concentration provoked cell death at 24 h of incubation in about one-third of T lymphocytes, NK cells and monocytes, and in about 60% of B cells, isolated from so-called CBD-sensitive subjects, which represented about one half of tested donors; for the other half, the effect of 20 μM CBD on PBMCs viability was insignificant [15]. In the present study, for the viability assays, we did not divide the donor group into CBD-resistant and CBD-sensitive subgroups, such that the extrapolated effect of 20 μM CBD on NK cell viability at 24 h (by about 15%, Figure 1B,C) was consistent with the result of the cited independent study.

Our study focused on the NK cells, so we addressed the mechanisms of CBD cytotoxicity as well as CBD’s suppression of NK cells’ killing potency, using leukemic Jurkat cells as a target. CBD (60 μM)-induced NK cell death could be partly prevented by CB_1_ but not by CB_2_ receptor antagonists (Figure 2). The latter might be somewhat surprising, bearing in mind that CB_2_ receptors are dominant over CB_1_ ones in immune cells, especially in NK and B cells [31]. Meanwhile, in mice, the CB_1_ receptor antagonist reverted the suppression of NK cell activity, induced by Δ^9^-Tetrahydrocannabinol, more efficiently than the CB2 receptor antagonist [32]. Therefore, the lower abundance of CB_1_ as compared to CB_2_ receptors may not be the crucial factor for immunomodulation by cannabinoids but rather the functional expression of components of non-overlapping signaling pathways, linked to each particular receptor. However, the most powerful reversal of CBD cytotoxicity in our case was achieved by CsA, which prevents mPTP formation (Figure 3A). Previously, for two immune cell models, namely, monocytes and T-ALL cells, the key mechanism of CBD toxicity was shown to be mediated by stable mPTP formation [11,12]. The present study extends this conclusion to another PBMCs subpopulation, NK cells. Here, we also demonstrated that mPTP formation, induced by an increasing concentration of CBD, provoked increased mitochondrial depolarization (Figure 3). Mitochondrial defunction is associated with decreased metabolic activity, increased ROS production and the release of apoptotic factors, as demonstrated previously [11,12]. More detailed studies on Jurkat cells have revealed that the primary target for CBD was a voltage-dependent anion channel, VDAC, in the outer mitochondrial membrane. The binding of CBD to VDAC promoted Ca^2+^ entry via this channel and mitochondrial Ca^2+^ overload, a cause of mPTP formation [11], whereas CBD-induced apoptosis was prevented by a selective inhibitor of VDAC oligomerization, but not by the CB_2_ receptor antagonist [33]. Of note, PBMCs and the NK cell subpopulation displayed less altered mitochondrial potential at high (60 μM) CBD concentrations than T-ALL (Jurkat) cells, where mitochondrial potential collapsed to almost zero (Figure 3). Interestingly, low (5 μM) CBD caused a larger decrease in mitochondrial potential in NK cells as compared to Jurkat cells. Yet, in contrast to its immediate effect, after 24 h with 5 μM CBD, a significant fraction of NK cells displayed an increased (by absolute value) ΔΨm (Figure 3E). This may be related to the fact that the moderate elevation of matrix Ca^2+^ stimulates mitochondrial metabolism, such that cells that better control their mitochondrial Ca^2+^ homeostasis by avoiding Ca^2+^ overload can take advantage of the Ca^2+^ entry induced by low doses of CBD [11].

The difference in NK cell functionality in different donors is clearly demonstrated by the data in Figure 4, showing a great variability in NK cell degranulation activity, stimulated by coculturing with Jurkat cells. Moreover, the degranulation activity of NK cells obtained from the same apparently healthy donor on different days varied significantly, indicating the dynamic nature of this process and its dependence on the individual’s current physical state. Possible reasons for such differences can include recent infections, stress levels, circadian rhythms, dietary factors, and other variables (reviewed in [34]). However, they are independent of the extent of degranulation response, which was not notably changed between 0 and 25 μM CBD pretreatments.

In contrast to the large variation in degranulation, NK cells from different donors showed comparatively high cytotoxicity against target cells (Figure 5B,C; cultures with NK cells not pretreated with CBD). This can be attributed to the fact that NK cells utilize two distinct killing modes: fast granule exocytosis and slower death receptor (FasL/CD95)-mediated apoptosis [30]. Of note, the Jurkat cells used in this study as target cells constitutively express high levels of CD95 [35]. On the other hand, excessive degranulation is not optimal, and its moderation can increase killing potency [36]. Therefore, killing potency is not proportional to degranulation. When it comes to the effect of CBD pretreatment on NK cells’ cytotoxicity against Jurkat cells, NK cells isolated from half of the donors demonstrated no significant change with the increase in CBD concentration from 0 to 25 μM, whereas in the other half of donors, the cytotoxicity was halved (Figure 5). Thus, while CBD did not affect the degranulation process at concentrations up to 25 μM, a reduction in killing efficiency was observed in some donors even when NK cells were pretreated with 10 μM CBD. This may be due to the fact that pretreatment with CBD can reduce granzyme content in the lytic granules of NK cells, as has recently been demonstrated [37]. Furthermore, it can be hypothesized that CBD can disrupt the functioning of the CD95 (Fas/FasL) signaling pathway; however, experimental data on this matter are currently lacking. It should also be noted that when comparing the immunosuppressive effects of the two major components of *Cannabis*, non-psychotropic CBD and psychotropic Δ^9^-Tetrahydrocannabinol, the latter appeared to be more potent [38].

As demonstrated in our earlier study, 25 μM CBD eliminated about 90% of T-ALL cells, but cancer cells of other types were much more resistant to CBD [11]. For instance, the IC_50_ for B-ALL at 24 h of incubation of the cells was four times higher compared to T-ALL cells (50–55 μM and 12–13 μM, respectively), and comparable to that reported here for NK cells (Figure 1B). The treatment of cancers with higher CBD concentrations will inevitably affect innate anticancer immunity. Therefore, in the case of each cancer type, its relative resistance to CBD should be determined. An attractive approach to avoiding the collateral effects of CBD, including lymphopenia induction, might involve an advanced formulation of CBD, aimed at reducing its efficient concentration and to targeting it specifically at cancer cells [1,39]. The use of CBD for palliative purposes can result in variable plasma concentrations, ranging from the low-nanomolar to the low-micromolar range, with peak concentrations of approximately 2 µM [1]. The results of the present study show that these concentrations are not toxic to NK cells and do not cause a decrease in their functionality. Thus, palliative CBD use seems to be compatible with NK cell-based immunotherapy.

## 5. Conclusions

Considering that NK cells are an important branch of antitumor immunity, NK cell- based immunotherapy is gaining significant interest as a promising and potentially safe therapeutic anticancer strategy. Allogenic healthy donor-derived NK cells represent the first-choice source for this therapy. Phytocannabinoids and particularly non-psychotropic CBD have received increased attention for use in palliative care for cancer patients, and have recently been considered as potential anti-cancer drugs. To evaluate the compatibility of NK cell-based immunotherapy and CBD use in cancer patients, the present study examined the effect of CBD on the viability and antitumor properties of primary human NK cells. We demonstrated that the pre-treatment of NK cells with CBD at concentrations of 0.5–5 µM did not affect their ability to induce the death of leukemic target cells. Higher concentrations (10–25 µM) reduced this capacity in a dose-dependent manner in half of tested donors, whereas for the other half, the NK killing ability was maintained up to 25 µM CBD. At the same time, CBD concentrations in the 30–100 µM range induced the death of primary human NK cells. This process was driven by mitochondrial depolarization, resulting from the formation of the mitochondrial permeability transition pore (mPTP) mechanism, which has been previously described in leukemic cells and freshly isolated monocytes. Based on our results, it can be concluded that the use of CBD in palliative care at doses resulting in plasma or serum concentrations below 5 µM is safe and does not impair NK cell functional activity. Regarding the incorporation of this compound into chemotherapy regimens, the tumor type must be considered. For tumors highly sensitive to CBD (e.g., T-ALL) it is unlikely that CBD would significantly impact the NK cell function. Conversely, when treating less CBD-sensitive tumors, the use of high CBD concentrations could adversely affect NK cell viability and functional activity, and therefore is not recommended.

## Data Availability

Data are contained within the article.

## Abbreviations

The following abbreviations are used in this manuscript:

NK	Natural killer
CBD	Cannabidiol
CsA	Cyclosporine A
PBMC	Peripheral blood mononuclear cells
TRPV	Transient Receptor Potential (channel) from Vanilloid subfamily
CB1/2	Cannabinoid (receptor) type 1/2
mPTP	Mitochondrial permeability transition pore
T-ALL	T-type acute lymphoblastic leukemia
PI	Propidium iodide
TMRE	Tetramethylrhodamine ethyl ester
IL2/15	Interleukin 2/15
ΔΨm	Voltage difference across the mitochondrial inner membrane
VDAC	Voltage-dependent anion channel
